# Precocious flowering of juvenile citrus induced by a viral vector based on *Citrus leaf blotch virus*: a new tool for genetics and breeding

**DOI:** 10.1111/pbi.12555

**Published:** 2016-03-25

**Authors:** Karelia Velázquez, Jesús Agüero, María C. Vives, Pablo Aleza, José A. Pina, Pedro Moreno, Luis Navarro, José Guerri

**Affiliations:** ^1^ Centro de Protección Vegetal y BiotecnologíaInstituto Valenciano de Investigaciones Agrarias (IVIA)MoncadaValenciaSpain

**Keywords:** citrus vector, speed up genetic studies, early flowering in juvenile citrus plant

## Abstract

The long juvenile period of citrus trees (often more than 6 years) has hindered genetic improvement by traditional breeding methods and genetic studies. In this work, we have developed a biotechnology tool to promote transition from the vegetative to the reproductive phase in juvenile citrus plants by expression of the *Arabidopsis thaliana* or citrus *FLOWERING LOCUS T* (*FT*) genes using a *Citrus leaf blotch virus*‐based vector (*clbvINpr‐AtFT* and *clbvINpr‐CiFT*, respectively*)*. Citrus plants of different genotypes graft inoculated with either of these vectors started flowering within 4–6 months, with no alteration of the plant architecture, leaf, flower or fruit morphology in comparison with noninoculated adult plants. The vector did not integrate in or recombine with the plant genome nor was it pollen or vector transmissible, albeit seed transmission at low rate was detected. The *clbvINpr‐AtFT* is very stable, and flowering was observed over a period of at least 5 years. Precocious flowering of juvenile citrus plants after vector infection provides a helpful and safe tool to dramatically speed up genetic studies and breeding programmes.

## Introduction

Breeding programmes for citrus, the main fruit crop worldwide, are necessary to obtain high‐quality competitive varieties and rootstocks. The complex reproductive biology of citrus trees due to apomixis, pollen and/or ovule sterility of several high‐quality genotypes and self‐ and cross‐incompatibility that are relatively common among citrus genotypes limits the possibilities to select parents for specific crosses (Ollitrault and Navarro, 2012). However, the most important impairment for breeding programmes and genetic studies is the long juvenile period of citrus, often lasting more than 6 years (Krajewski and Rabe, 1995). Plants in the juvenile phase are characterized by their vigorous growth, the presence of thorns and failure to flower even under inductive conditions; they must undergo a transition from the juvenile phase to the reproductive phase to develop flowering buds. Thus, the long juvenile period of citrus plants impairs for years the generation of segregating populations by back crossing, hybrid progeny evaluation to obtain new varieties or studies on inheritance of fruit‐related traits.

The genetic factors that control the end of the juvenile phase to start the flowering period in citrus are presently unknown. Recent molecular studies suggested that the FLOWERING LOCUS T (FT) protein in *Arabidopsis thaliana* and its orthologs in other plant species are the primary component of the flowering signal cascade (Corbesier *et al*., 2007; Endo *et al*., 2005; Hecht *et al*., 2011; Imamura *et al*., 2011; Jaeger and Wigge, 2007; Kong *et al*., 2010; Lin *et al*., 2007; Lv *et al*., 2012; Tamaki *et al*., 2007). In the shoot apical meristem, the FT protein forms a complex with the FD protein (with a basic domain/leucine zipper motif transcription factor), which up‐regulate the gene *APETALA 1 (AP1*) in the meristem to induce an inflorescence (Abe *et al*., 2005; Wigge *et al*., 2005). In Satsuma (*Citrus unshiu* (Macf.) Marc.) and Moncada mandarins (*C. clementina* Hort. ex Tan. × (*C. unshiu* × *C. nobilis* Lour.)), in which floral induction is triggered by low temperatures during the fall and winter, the messenger RNA (mRNA) level of the citrus ortholog of *FT* (*CiFT*) increases during the spring just before blooming (Muñoz‐Fambuena *et al*., 2011; Nishikawa *et al*., 2007, 2009). Similarly, in trifoliate orange (*Poncirus trifoliata* (L.) Raf.), in which floral induction and flower bud development occur during early summer as in many deciduous trees, the amount of *CiFT* mRNA increases in early summer (Nishikawa *et al*., 2009). Ectopic expression of *CiFT* under the control of the 35S promoter of *Cauliflower mosaic virus* (CaMV 35S) induces an early flowering phenotype on trifoliate orange (Endo *et al*., 2005); however, overexpression of *FT* also affects the growth and architecture of these plants that show a dwarfed aberrant appearance. Precocious flowering was also observed in juvenile transgenic plants expressing the flowering genes *LEAFY* and *APETALA1* (Peña *et al*., 2001), albeit plants expressing LEAFY also showed an abnormal phenotype. Additionally, recovery of transgenic citrus plants is usually slow and difficult.

Plant virus vectors have been used for both expression of foreign genes (Gleba *et al*., 2007) and suppression of endogenous target genes by virus‐induced gene silencing (VIGS) in the infected plants (Senthil‐Kumar and Mysore, 2011). Using viral vectors is a quick system to deliver proteins in plants without any need for the transformation and regeneration techniques required to obtain transgenic plants. Moreover, expressing the FT protein with viral vectors is an emerging technology to induce precocious flowering in plants (McGarry and Ayre, 2012; Yamagishi *et al*., 2014).

*Citrus leaf blotch virus* (CLBV), the type member of the genus *Citrivirus*, family Betaflexiviridae (Adams *et al*., 2012; Martelli *et al*., 2007), has filamentous virions about 960 × 14 nm in size composed of a single‐stranded, positive‐sense genomic RNA (gRNA) of 8747 nucleotides (nt) and a 41‐kDa coat protein (CP) (Galipienso *et al*., 2001; Vives *et al*., 2001). The CLBV gRNA has three open‐reading frames (ORFs) and untranslated regions of 73 and 541 nt at the 5′ and 3′ ends, respectively. The ORF1, encoding a ~227‐kDa replicase polyprotein, is translated directly from the gRNA. The ORF 2, encoding a ~40‐kDa protein with a motif characteristic of cell‐to‐cell movement proteins (MP) of the 30K superfamily, and the ORF3, encoding the CP, are translated from two 3′ co‐terminal subgenomic RNAs (MP sgRNA and CP sgRNA, respectively). ORFs 2 and 3 are separated by a 64‐nt intergenic region (Renovell *et al*., 2010; Vives *et al*., 2001, 2002a). Different viral vectors based on a full‐genome infectious cDNA clone of CLBV (Vives *et al*., 2008a) were previously obtained (Agüero *et al*., 2012, 2014). The advantages of CLBV to be used as a viral vector for citrus are as follows: (i) it causes a symptomless infection in most commercial cultivars (Galipienso *et al*., 2000); therefore, phenotype changes due to endogenous gene silencing or foreign gene expression would not be masked by virus symptoms, (ii) because CLBV is not restricted to the phloem, these vectors are appropriate for gene expression or silencing in nonphloem tissues including meristematic regions (Agüero *et al*., 2013), and (iii) contrarily to *Citrus tristeza virus* (CTV), also used as viral vector for citrus (Folimonov *et al*., 2007), CLBV is not transmitted by vectors and therefore it could be safely used in field experiments.

In this work, we have developed a biotechnology tool to promote an early transition to flowering of juvenile citrus plants by expression of the *A. thaliana* or citrus *FT* gene in the *clbvINpr* vector (*clbvINpr‐AtFT* or *clbvINpr‐CiFT* vectors, respectively*)*. Graft inoculation of juvenile citrus plants with either of these vectors induced flowering in the second flush [about 4 months postinoculation, (mpi)]. The vector *clbvINpr‐AtFT* was very stable, and flowering of the vector‐inoculated plants was observed in successive flushes over a period of at least 5 years. Precocious flowering after vector infection provides a helpful tool to dramatically speed up genetic studies and breeding programmes.

## Results

### Induction of flowering in juvenile citrus plants by means of a CLBV‐based vector expressing the *FT* gene

**Figure 1:**
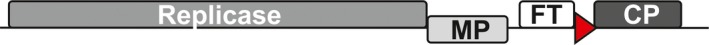
Outline of the *clbvlNpr‐AtFT* or *clbvlNpr‐CitFT* vectors generated by cloning the *FT* gene from *Arabidopsis. thaliana* or Valencia late sweet orange (*Citrus sinensis* (L.) Osb.), respectively, in the *ClbvINpr* vector. Triangle represents the duplicated CP sgRNA promoter. White box represents the *FT* gene introduced.

**Figure 2:**
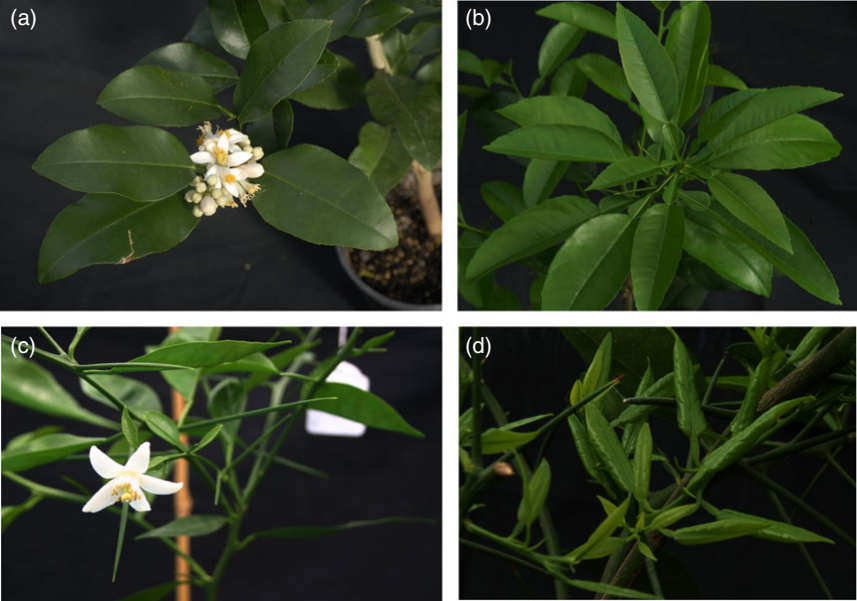
Flowering of a juvenile *C. excelsa* (Wester) plant (a), and a triploid hybrid recovered from a hybridization between a tetraploid Fina clementine (*C. clementina* Hort. ex Tan.) and a diploid tangor hybrid (*C. clementina × C. sinensis*) (c) inoculated with the *ClbvINpr‐AtFT* viral vector, respect to the same genotype inoculated with the wild‐type CLBV virus (Figure 2b,d, respectively) propagated on Rough lemon (*Citrus jambhiri* Lush) rootstock.

To investigate whether ectopic expression of the *FT* gene from a CLBV vector is able to induce flowering in infected juvenile citrus plants, a cDNA of the *FT* gene from *A. thaliana* or from Valencia late sweet orange (*Citrus. sinensis* (L.) Osb.) was cloned into the *clbvINpr* vector to generate the *clbvINpr‐AtFT* and *clbvINpr‐CiFT* constructs, respectively (Figure 1) (see ‘Experimental procedures’). These constructs were agroinoculated in *Nicotiana benthamiana* plants, and 1 month later, the resulting recombinant virions were purified and slash inoculated in two juvenile *C. excelsa* Wester seedlings. At the end of the second flush, about 4 mpi, the citrus plants infected with both constructs flowered (Figure 2a). No differences were found in either the number of flowers or in the period of flowering between the plants inoculated with the vectors expressing *AtFT* or *CiFT*. Later, lateral shoots started growing until the formation of a new floral bud. Some shoots did not produce flowers, may be due to uneven distribution or accumulation of the recombinant virus in host plants (Agüero *et al*., 2014). As the constructs are very stable, flowering and fruit set occurred in successive flushes during the observation period (at least 5 years). Similar *C. excelsa* plants noninoculated or inoculated with the wild‐type CLBV virus (WT‐CLBV) (Figure 2b) did not produce flowers, but continued their vegetative growth for the same period.

**Figure 3:**
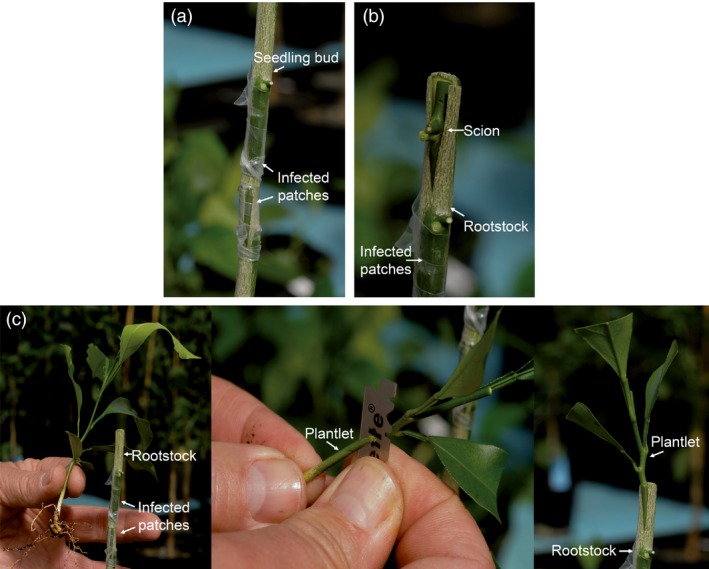
Graft inoculation procedures used to infect citrus plants with the *clbvINpr‐FT* viral vector to induce early flowering: (a) graft inoculation of seedling plants of the candidate genotype with two infected bark patches and then decapitation of the plant leaving one bud above the inocula, (b) propagation of a scion bud of the candidate genotype onto a Rough lemon rootstock with simultaneous graft inoculation of the rootstock with two bark patches from a plant infected with the viral vector, and (c) elimination of the root of a small plantlet of the candidate genotype and top‐working the upper part on a Rough lemon rootstock with simultaneous graft inoculation of the viral vector as in (b).

The *C. excelsa* plants infected with the *clbvINpr‐AtFT* or the *clbvINpr‐CiFT* constructs were used as inoculum sources for other citrus hybrid juvenile plants obtained in the framework of a citrus breeding programme (Navarro *et al*., 2015), following three different inoculation procedures: (i) seedling plants were graft inoculated with two infected bark patches and then cut back leaving one bud above the inocula (Figure 3a), (ii) buds from the juvenile hybrids were propagated onto a rootstock (e.g. a Rough lemon (*C. jambhiri* Lush)), simultaneously graft inoculated with two bark patches from the infected *C. excelsa* plants (Figure 3b), and (iii) the root of small hybrid plantlets was cut‐off at the crown and the aerial portion was top‐worked onto a rootstock, simultaneously graft inoculated with two bark patches from the infected *C. excelsa* plants (Figure 3c). All plants inoculated by either procedure with the *clbvINpr‐AtFT* or the *clbvINpr‐CiFT* constructs became systemically infected, as detected by conventional reverse transcription PCR (RT‐PCR) (Galipienso *et al*., 2004), using primers flanking the insertion site of the *FT* gene in the vector (Agüero *et al*., 2014). Some genotypes flowered in successive flushes along the year, whereas others only flowered in the spring flush, suggesting that factors other than the FT protein may be necessary to induce flowering. Indeed no correlation was observed between flowering intensity and CLBV accumulation as estimated by RT quantitative real‐time PCR (RT‐qPCR) (Ruiz‐Ruiz *et al*., 2009) (data not shown).

The expression of the FT protein did not affect manifestation of other citrus juvenile characters like thorniness (Figure 2c). No difference in thorn size or abundance was observed between plants noninoculated or inoculated with the WT‐CLBV (Figure 2d) or inoculated with either the *clbvINpr‐AtFT* or the *clbvINpr‐CiFT* constructs (Figure 2c).

### Inoculation with *clbvINpr‐AtFT* did not alter plant architecture or flower and fruit characteristics

**Figure 4:**
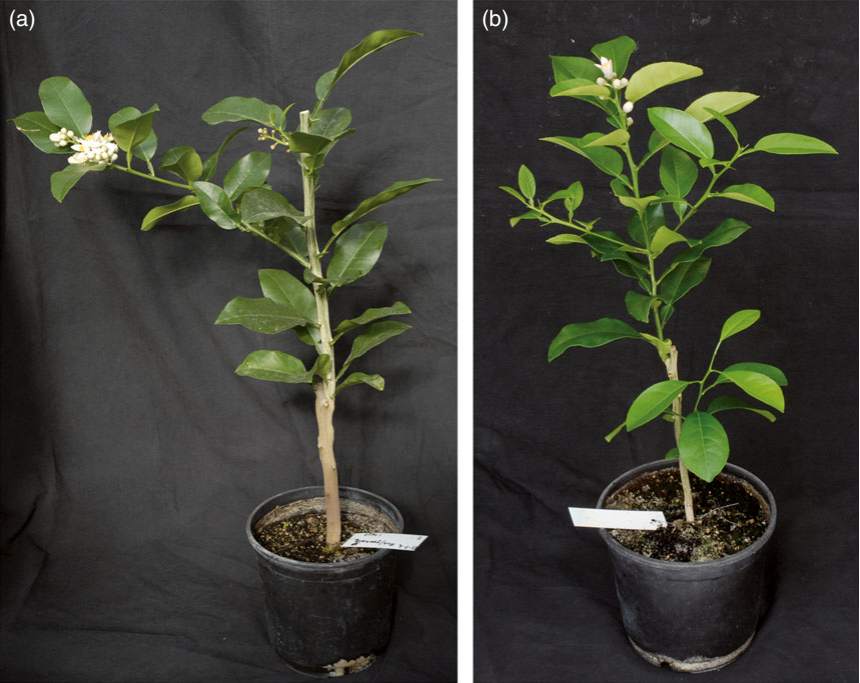
Plant architecture of (a) a noninoculated adult *C. excelsa* plant and (b) a juvenile *C. excelsa* plant inoculated with the *clbvINpr‐AtFT* viral vector. Both plants were prepared by propagation of healthy *C. excelsa* buds onto Rough lemon rootstocks.

The appearance of juvenile plants inoculated with *clbvINpr‐AtFT* was similar to that of nonjuvenile plants, with no leaf deformation or stunting observed in any citrus genotype infected with the recombinant virus (Figure 4b).

To confirm the above observation, buds from juvenile and adult plants of *C. excelsa* and Cleopatra mandarin (*C. reshni* Hort. ex Tan.) were propagated on Rough lemon rootstock and the plants propagated with buds from juvenile plants were simultaneously graft inoculated with *clbvINpr‐AtFT*. During the first year, only the juvenile plants inoculated with the viral vector flowered, whereas in the second year, flowering occurred in both the juvenile *clbvINpr‐AtFT*‐infected plants and the adult noninoculated plants. The juvenile *clbvINpr‐AtFT*‐infected plants showed the same architecture as the adult plants, with no dwarfing of leaf deformation being observed (Figures 4a,b). The adult and the *clbvINpr‐AtFT*‐infected juvenile *C. excelsa* plants usually developed inflorescences (Figures 4a,b), whereas the Cleopatra mandarin plants produced individual flowers at the end of the shoots.

**Figure 5:**
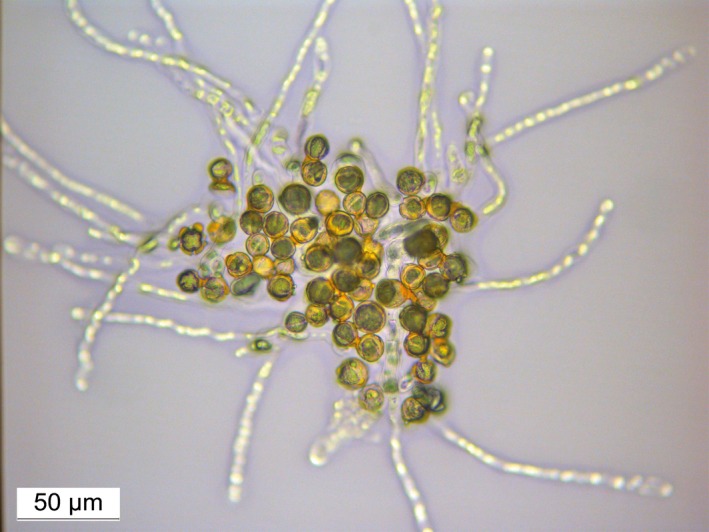
Germination of pollen from a juvenile *C. excelsa* plant inoculated with the *clbvINpr‐AtFT* viral vector.

**Figure 6:**
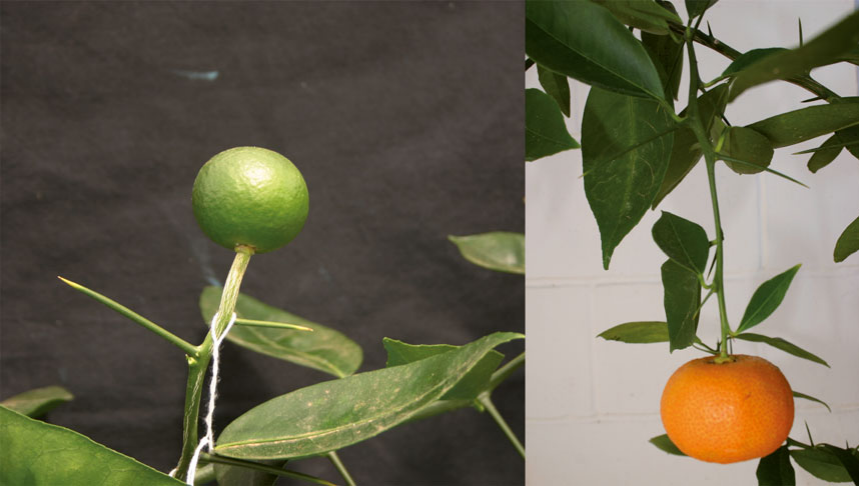
Fruit development in a triploid hybrid obtained by hybridization between a tetraploid Fina clementine and a diploid tangor. A triploid bud was propagated onto a Rough lemon rootstock inoculated with the *clbvINpr‐AtFT* viral vector.

**Figure 7:**
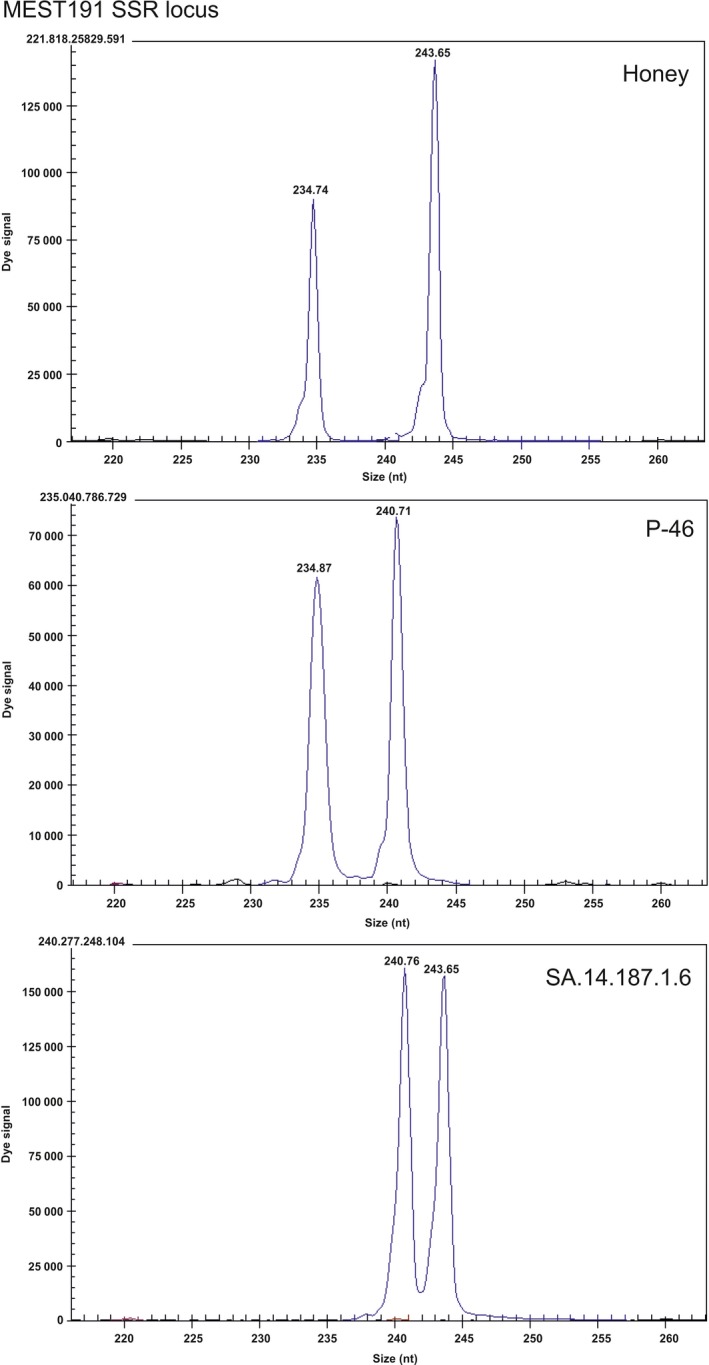
Electropherograms obtained using the MEST191 SSR locus for genetic analysis of a diploid Honey hybrid mandarin (*Citrus nobilis* × *Citrus deliciosa*), the P‐46 diploid tangor hybrid [(*Citrus reticulata *× *Citrus sinensis*) × *Citrus sinensis*)] and the triploid hybrid SA.14.187.1.6, displaying the alleles of both parents. The numbers indicate the size of alleles in nucleotides for each SSR locus.

The floral morphology and the floral organs, such as sepals, petals, stamens or pistil, were normal in all juvenile *clbvINpr‐AtFT*‐infected plants. Pollination of pistils from juvenile *clbvINpr‐AtFT* inoculated plants with pollen from adult plants of other citrus genotypes led to fruit set. The fruits developed normally and their size, skin colour and aroma were indistinguishable from those developed on adult plants. The fruit yield of both adult and juvenile *clbvINpr‐AtFT*‐infected plants was similar. Pollen from precocious flowers germinated on agar plates (Figure 5) and successfully fertilized flowers of adult plants of other citrus genotypes with subsequent fruit development and maturation, also indistinguishable from normal fruits of female adult plants (Figure 6). Fruits of these latter plants were collected when ripe and seeds were germinated. Forty‐nine recovered plants were analysed with five Simple Sequence Repeat (SSR) markers (Froelicher *et al*., 2008; Ollitrault *et al*., 2012) and genetic analysis confirmed that all plants were hybrids of the parental genotypes used (Figure 7).

### Genetic stability of the *clbvINpr‐AtFT* vector

Genetic stability of the *clbvINpr‐AtFT* vector was examined by RT‐PCR analysis of total RNA (RNAt) from the two originally infected *C. excelsa* plants and from 10 other citrus genotypes graft inoculated from them, using primers encompassing the insertion site of the *AtFT* gene in the CLBV genome. This analysis, carried out more than 4 years after inoculation, showed in nine of the 12 plants a unique band of the size expected for the full *FT* sequence, whereas in the other three plants an additional weak band of smaller size was also observed. To quantify the recombination events, the PCR products were cloned and 422 individual clones were analysed by PCR using the same primers. Only 36 of these clones (8.5%) had an insert of a size smaller than that expected for the complete *AtFT* gene. To assess the sequence variability in the population, four small‐sized and 156 full‐sized clones were sequenced. The four small‐sized clones showed an identical deletion between the nucleotides 351 and 462 of the *AtFT* gene, suggesting that these nucleotides could be a hot spot for recombination. Also, as expected for a RNA virus population, nucleotide changes were observed in 90 of the 156 full‐sized clones sequenced (54 clones with a single nt change, 27 with two and 9 with three), with about 90% of these mutations involving an amino acid change. Overall, these results indicate that 4 years after inoculation there were few important changes in the *AtFT* sequence and the functionality of FT protein remained unaffected.

### Expression of flowering‐related genes in citrus plants inoculated with the *clbvINpr‐AtFT* vector

**Figure 8:**
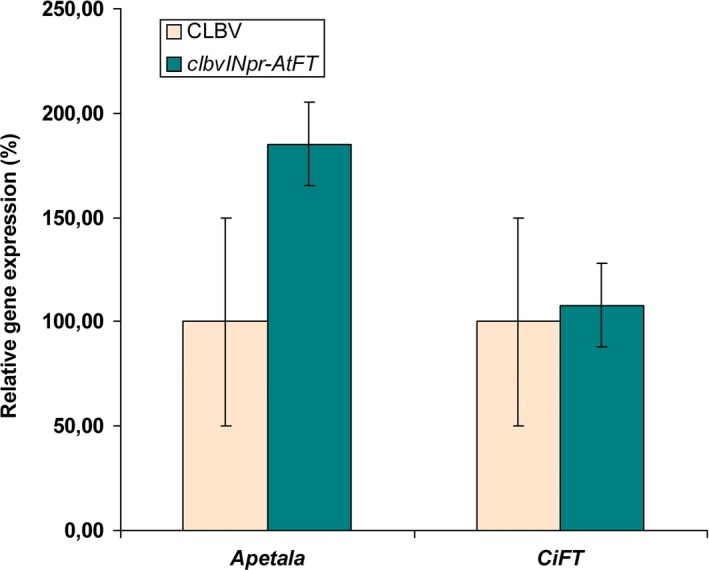
Relative mRNA expression of the endogenous *APETALA* (*AP*1) and citrus *FLOWERING LOCUS* T (*CiFT*) genes in plants of *C. excelsa* propagated on Rough lemon rootstock and inoculated with the *clbvINpr‐AtFT* vector or with WT‐CLBV. The mRNA levels were estimated by RT quantitative real‐time PCR. The average expression in control plants infected with WT‐CLBV was considered 100, and the expression in the *clbvINpr‐AtFT*‐infected plants was relative to the average of the control plants. Bars represent standard deviation values.

To examine how the presence of *AtFT* in infected citrus plants affected the expression of flowering‐related genes, buds of *C. excelsa* juvenile plants were propagated on ten Rough lemon seedling rootstocks, five of which were simultaneously graft inoculated with the *clbvINpr‐AtFT* vector, and five with the WT‐CLBV as control. At the end of the second flush, RNAt from a pool of shoot tips (about 10 mm in length) from each plant was purified and used to quantify the mRNA expression of the *AP*1 and *CiFT* citrus genes by RT‐qPCR. While significant differences in mRNA accumulation of the *AP*1 gene were observed (ANOVA, *P* < 0.05) in the shot tips of plants inoculated with *clbvINpr‐AtFT* in comparison with similar tissues from plants infected with WT‐CLBV, no significant difference (*P* > 0.05) was observed in the mRNA levels of the *CiFT* gene (Figure 8). These results indicate that the expression of *AtFT* in infected plants did not trigger silencing of the endogenous *CiFT* gene, probably due to the low sequence identity (74%) between *AtFT* and *CiFT* and the low *CiFT* mRNA expression in shot tips of juvenile citrus plants.

### Horizontal and vertical transmission of the *clbvINpr‐AtFT* vector

To assess whether the *clbvINpr‐AtFT* vector or a part thereof can become integrated in the plant genome, total DNA was isolated from bark and leaves of infected *C. excelsa* plants and analysed by PCR using primers designed on different parts of the CLBV genome or on the *AtFT* gene. No DNA amplification was ever obtained with CLBV‐ or *AtFT*‐specific primers, indicating that neither of them become integrated into the plant genome. Also, to examine potential recombination between the *AtFT* gene expressed from the viral vector and the endogenous *CiFT* gene, the DNA band PCR amplified with primers specific for the *CiFT* gene was cloned and 10 of these clones were sequenced. All of them had the characteristic sequence of *CiFT,* and no recombination event was detected.

In a previous experiment, seed transmission of CLBV was observed at a rate of 2.5% (Guerri *et al*., 2004). To examine whether the virus could be transmitted by pollen, we analysed RNAt from pollen from *clbvINpr‐AtFT‐*infected plants by RT‐qPCR with CLBV‐specific primers (Ruiz‐Ruiz *et al*., 2009), but no amplification was detected. Moreover, cross‐pollination of flowers from noninoculated plants with pollen from *clbvINpr‐AtFT‐*infected plants and further analysis of the offspring for the presence of CLBV resulted in none of the progeny plants being infected by the viral vector. Finally, to assess whether *clbvINpr‐AtFT* can be ovule‐transmitted, 125 seeds from different infected genotypes cross‐pollinated with pollen from healthy plants were grown and individual seedlings were analysed for CLBV by RT‐PCR. Only in one of the plants analysed was *clbvINpr‐AtFT* detected, confirming the low seed transmission rate previously detected for CLBV.

## Discussion

The juvenile phase characteristic of some woody perennials like citrus has been a limiting factor for traditional breeding and genetic studies. Expression of the *FT* gene from *A. thaliana* (*AtFT*) or citrus *(CiFT)* by means of a CLBV‐based viral vector induced precocious flowering in juvenile citrus plants. When a juvenile citrus plant was graft inoculated with the *clbvINpr‐AtFT* or the *clbvINpr‐CiFT* vectors, the infected plant started flowering within the next 4–6 months depending on the genotype and time of the year, whereas similar citrus plants noninoculated or infected with WT‐CLBV did not flower, but remained vegetatively growing under the same conditions. No difference in the juvenile plant response was observed using the *clbvINpr‐AtFT* or the *clbvINpr‐CiFT* vectors; however, to prevent a potential interference of endogenous *CiFT* silencing induced by the second vector, the *clbvINpr‐AtFT* construct was preferred in most of the experiments. These results suggest that the FT protein delivered by the viral vector could terminate the vegetative growth by inducing the transition from vegetative to reproductive meristematic tissues in the shoot apex. The ability of CLBV to invade meristematic regions in citrus (Agüero *et al*., 2014) may favour delivery of the FT protein in this region. Previous studies on expression of flowering‐related genes in the shoot apex of mature citrus trees found increased *CiFT* mRNA levels upon transition from the vegetative to the reproductive growth state (Muñoz‐Fambuena *et al*., 2011; Nishikawa *et al*., 2009). Similar changes might be induced in the shoot apex of plants inoculated with the viral vector. Up‐regulation of the floral meristematic gene *AP*1 in shoot tips of plants inoculated with the viral vector in comparison with plants noninoculated or inoculated with WT‐CLBV supports this hypothesis.

Constitutive expression of *CiFT* in transgenic *P. trifoliata* plants also induced early flowering, but it was accompanied by phenotypic aberrations consisting of a dwarfed, highly branched growth, misshapen leaves and altered flowering habit and flower morphology in comparison with the nontransgenic controls (Endo *et al*., 2005; Nishikawa *et al*., 2010). Contrastingly, in plants inoculated with either the *clbvINpr‐AtFT* or the *clbvINpr‐CiFT* constructs, the plant architecture was not affected and leaves, flowers and mature fruits were normal. This different behaviour could be due to (i) the slow accumulation and movement of CLBV in the inoculated plants that would allow normal growth during the initial period, before reaching a threshold virus concentration (and FT expression) necessary to induce flowering and (ii) the uneven accumulation and limited virus titre in fully infected plants that would provide reduced amounts of the FT protein in comparison with transgenic plants expressing FT in all their cells. Several observations support the slow accumulation and movement of CLBV: (i) in plants inoculated with a *clbvINpr* vector expressing the green fluorescent protein (*clbvINpr*‐GFP), CLBV was detected by RT‐PCR only at the end of the first flush (about 1.5 mpi) (Agüero *et al*., 2012), (ii) in plants inoculated with CLBV‐based vectors carrying fragments of the endogenous citrus genes *phytoene desaturase* or *sulphur*, the silencing phenotype was rarely observed in the first flush and more usually in the second flush, always being more intense in the second flush, and (iii) the symptoms of chlorotic blotching and stem pitting induced by CLBV in Dweet tangor (*C. tangerina* Hort. ex Tan. × *C. sinensis*) and Etrog citron (*C. medica* L.), respectively, usually appear in the second flush, not in the first. Finding that while some citrus genotypes infected with *clbvINpr‐AtFT* produced inflorescences, others produced individual flowers, or that some genotypes flowered only in the spring, whereas others repeatedly flowered in successive flushes along the year suggest that, in addition to the *FT* gene, other factors are likely necessary to induce floral buds.

Genetic instability associated with frequent recombination events and high mutation frequency during RNA replication are limiting factors in the use of plant viral vectors. However, *clbvINpr‐AtFT* was able to induce flowering during the long period studied (almost 5 years). PCR analysis of individual *AtFT* clones showed that only 8.5% of the clones analysed yielded a DNA band of a size smaller than that expected for the complete gene, confirming that CLBV‐based vectors are highly stable (Agüero *et al*., 2012, 2014). Although some of the clones sequenced showed nucleotide substitutions leading to amino acid changes, none of these changes affected the amino acids essential for the FT function (Hanzawa *et al*., 2005; Ho and Weigel, 2014). On the other hand, genetic variation of CLBV isolates from different geographical origin or citrus host was shown to be very low in comparison with most RNA plant viruses (Vives *et al*., 2002b).

Although genetic transformation to induce early flowering could be a helpful tool to accelerate breeding, this technique is less practical with citrus trees due to their low transformation efficiency and the long regeneration period required (Peña *et al*., 2001). While infection of a citrus plant with the viral vector is fast (about 1 month), recovery of a transgenic citrus plant takes more than 1 year. Also, once the viral vector has systemically infected a citrus plant, it can be easily graft inoculated onto hundreds of different genotypes, whereas the transformation protocol has to be adapted and performed for each individual genotype. The juvenile plants inoculated with CLBV‐based vectors expressing the *FT* gene produced flowers at the end of the second flush (about 4 mpi, depending on the inoculated genotype and the season) and these successfully produced fruits with normal seeds. Therefore, this technique allows obtaining seeds for the next‐generation progeny within 1 year without any genome modification in the plant.

The viral vector developed in this work is being a helpful tool to speed up genetic studies and breeding programmes. While backcrossing has not been used in citrus breeding because the very long time needed to recover successive hybrid populations, now it has been routinely incorporated in our triploid breeding programme (Navarro *et al*., 2015). Tetraploid parents were produced by sexual hybridization and screened by molecular markers for some desired traits, for example resistance to the fungus *Alternaria alternata* (Cuenca *et al*., 2013) or expression of the *Ruby* gen for accumulation of anthocyanins in fruits (Butelli *et al*., 2012). Then, selected hybrids are inoculated with the *clbvINpr‐AtFT* vector to induce precocious flowering and used in crosses with diploid parents to produce triploid progenies. More than 1600 sexual crosses have been made so far following this procedure.

In many countries, the use of genetically modified plants is presently prohibited. The main concerns against the transgenic plants are the use of antibiotic resistance genes for selection of transformants, potential cross‐pollination with other sexually compatible plants, plant genome alteration by integration of foreign sequences or by recombination when additional copies of endogenous genes are introduced, and potential transduction of the binary plasmid used for transformation into other bacteria present in the plant or soil. Inoculation of CLBV‐based vectors to citrus trees to induce early flowering is performed by grafting, and no antibiotic resistance gene, plasmid or bacteria are introduced in the plants. Neither the viral vector nor the *FT* gene introduced in the plants become integrated in the plant genome, and the CLBV virus is not transmitted either by insect vectors (Vives *et al*., 2008b) or by pollen. Although a low seed transmission rate has been detected in a few genotypes, the virus is easily detected in the infected plants by RT‐qPCR and it could be easily eliminated by shoot tip grafting *in vitro* (Navarro *et al*., 1975) to recover any desired genotype vector free. Overall, our results indicate that this viral vector should not be considered as a risky genetically modified organism and it can be transiently used under field conditions, with essentially no risk, in breeding programmes for early fruit evaluation even under field conditions. This will allow a quick screening of hybrid progeny to eliminate the genotypes with undesirable characters, which are the majority of the progeny in any breeding programme, and concentrate efforts in additional evaluation of selected potentially new varieties. Finally, selected varieties could be recovered vector free for commercial propagation. This approach will save expensive greenhouse space and labour and will accelerate the selection of new varieties, making the breeding programmes much more efficient at lower cost. A field experiment has been recently established in our institute for early evaluation of a triploid hybrid progeny inoculated with the *clbvINpr‐AtFT* vector.

Citrus breeding is also hampered by the scarcity of genetic studies on the inheritance of characters that cannot be phenotyped during the juvenile phase (Ollitrault and Navarro, 2012). The use of CLBV‐based vectors, together with the new genetic (Ollitrault *et al*., 2012) and genomic (Wu *et al*., 2014) tools available, will facilitate these studies allowing the development of markers for focused introgression and early selection. These new tools will enable general implementation of efficient breeding programmes based on marker‐assisted selection.

## Experimental procedures

### Construction of the CLBV‐based vectors expressing the *FT* gene

The *AtFT* and *CiFT* coding regions were amplified by RT‐PCR from *A. thaliana* and Valencia late sweet orange RNAt extracts using appropriate primers (Table 1) and inserted into the *Pml*I restriction site of the *clbvINpr* vector (Agüero *et al*., 2012) to generate the *clbvINpr‐AtFT* and *clbvINpr‐CiFT* constructs, respectively (Figure 1). The *clbvINpr* vector was built by introducing a unique *Pml*I restriction site at the intergenic region between the MP and the CP genes and then introducing a duplicate of the CP sgRNA promoter restoring the *Pml*I restriction site to express foreign sequences by producing an extra sgRNA. In this vector, transcription of the new sgRNA was induced by the native promoter of de CP sgRNA, whereas the CP gene expression was controlled by the duplicated promoter (Agüero *et al*., 2012, 2014). All the constructs were confirmed by sequencing.

**Table 1:** Primers used in this work

Gene synthesized	Primer	Sequence 5′–3′	Position (nt)
AtFT	AtFT F^a^	ATGTCTATAAATATAAGAGACCCTCTTATAGTAAGCAG	70–107^b^
	AtFT R^a^	CTAAAGTCTTCTTCCTCCGCAGCCACTCTCC	597–567^b^
CiFT	cFT F^a^	ATGTCTAGCAGGGAGAGAGACCCTCTTATTG	117–147^c^
	cFT R^a^	TCATCGTCTGACAGGCCTTCCG	650–629^c^
*AP1* q‐PCR	qAP1F	ATCAAATTCAAAACCAGGTTCCCAACA	740–766^d^
	qAP1R	ATCCAGCAAAGCATCCAAGGCTACAC	910–885^d^
*CiFT* q‐PCR	qCiFTF	ATGTCTAGCAGGGAGAGAGACCCTCT	117–142^c^
	qCiFTR	ATTAACATCCTTGTTTGAATAGGTAAT	204–230^c^

a Phosphorylated at the 5′ end.

b Nt positions are indicated on the sequence of the *Arabidopsis thaliana FT* (FLOWERING LOCUS T) mRNA (GenBank accession number AB027504).

c Nt positions are indicated on the sequence of the *Citrus unshiu* flowering locus CiFT2 mRNA (GenBank accession number AB301934).

d Nt positions are indicated on the sequence of the *Citrus clementina* hypothetical protein (CICLE_v10032490 mg) mRNA (GenBank accession number XM_006437813.1).

### Plant growth and inoculations

*N. benthamiana* plants were grown in small pots with an artificial potting mix (50% vermiculite and 50% peat moss) in a plant growth chamber at 20/24 °C (night/day), 60% relative humidity and a 16/8‐h light/dark regime. Citrus plants were grown as seedlings, or propagated on a Rough lemon rootstock, in a glasshouse at 18/26 °C (night/day), using 2‐L plastic containers filled with 50% sand and 50% peat moss and a standard fertilizing procedure (Arregui *et al*., 1982).

The *ClbvINpr‐AtFT* and *clbvINpr‐CiFT* constructs were agroinoculated into *N. benthamiana* using *Agrobacterium tumefaciens* cells, strain COR 308 (kindly provided by Dr. C. M. Hamilton, Cornell Research Foundation). Once confirmed the systemic infection, semipurified virion extracts from *N. benthamiana*‐infected plants (Galipienso *et al*., 2000) were mechanically inoculated onto *C. excelsa* plants by stem slashing (Garnsey *et al*., 1977) with scalpel blades dipped in the virion extracts (Galipienso *et al*., 2000; Vives *et al*., 2008a). Bark pieces from these plants or from plants infected with the WT‐CLBV were used to graft inoculate other citrus species or hybrids.

### RNA extraction, RT‐PCR detection and real‐time RT‐qPCR quantification

For RT‐PCR amplification and detection, RNAt from 100 mg fresh tissue was prepared using the TRIzol reagent (Invitrogen, Carlsbad, CA) following the manufacturer's instructions, and after alcohol precipitation, RNA was resuspended in 50 μL of RNase‐free water.

The *FT* sequences inserted in the *clbvINpr* vector were detected by conventional RT‐PCR (Vives *et al*., 2002b) using the primer pair MpC/MP3U (Agüero *et al*., 2014), flanking the insertion site. The DNA synthesized was analysed by 2% agarose gel electrophoresis and GelRed staining (Biotium Inc., Hayward, CA). The DNA fragments obtained were inserted into the pGEM‐T plasmid vector (Promega Corp. Madison, WI) and cloned in *Escherichia coli* DH5a cells. A total of 422 individual clones were analysed by PCR using the same primers, and the nucleotide sequence of 160 clones was determined with an ABI PRISM DNA Sequencer 3100 (Applied Biosystems, Paisley, UK). Multiple alignments were performed using the program Clustal W (Thompson *et al*., 1994).

To assess the endogenous gene expression by RT‐qPCR, RNAt from young shoots from noninoculated or vector‐infected plants was prepared using a standard protocol with two phenol: chloroform: isoamyl alcohol extractions, followed by RNA precipitation with 6 m lithium chloride (Ancillo *et al*., 2007). RNAt extracts were treated with RNase‐free DNase (Turbo DNA‐free; Ambion, Paisley, UK). RNAt concentrations were measured in duplicate in a NanoDrop 1000 UV–VIS spectrophotometer (Thermo Fisher Scientific, Waltham, MA) and then adjusted to the same concentration. DNA‐free RNAt from plants infected with the WT‐CLBV or the *clbvINpr‐AtFT* constructs was reverse‐transcribed using oligo(dT) and ThermoScript^™^ reverse transcriptase (Invitrogen). Apetala (*AP1*) and *CiFT* mRNA levels were estimated by RT‐qPCR using SYBR GREEN detection and specific primers (Table 1). The relative mRNA levels were normalized to the RNAt amounts as previously described by Agüero *et al*. (2012). The expression of each gene in the *clbvINpr‐AtFT*‐inoculated plants relative to the control WT‐CLBV‐infected plants was determined by the 2^−ΔΔCT^ method (Livak and Schmittgen, 2001). These values were subjected to multifactorial analysis of variance (ANOVA) using the IBM SPSS Statistics 20.0 program.

### Genotyping the progeny by SSR marker analysis

The male and female parents and the 49 hybrids selected were genotyped using a total of five SSR markers: CIBE5720 (Ollitrault *et al*., 2010), MEST191, (Ollitrault *et al*., 2012), MEST488 (García‐Lor *et al*., 2012), mCrCIR01F04a (Froelicher *et al*., 2008) and mCrCIR07F11 (Kamiri *et al*., 2011). Twenty‐eight of the triploid hybrids were analysed from a 4x × 2x sexual hybridization between a tetraploid Temple mandarin (*C. temple* Hort. ex Y. Tan.) and the P‐48 diploid hybrid ((*C. reticulata* × *C. sinensis*) × *C. sinensis*), and the other 21 triploid hybrids from a 2x × 2x sexual hybridization between Honey mandarin (*C. nobilis* × *C. deliciosa* Blanco) and the P‐46 diploid hybrid ((*C. reticulata* × *C. sinensis*) × *C. sinensis*). In both hybridizations, the male parents were inoculated with the *clbvINpr‐AtFT* viral vector.

Genomic DNA was isolated from the hybrids and their parent genotypes using the Plant DNAeasy kit (Qiagen, Hilden, Germany), following the manufacturer's protocol. PCR amplification of the genomic DNA with the SSR markers was performed using a Mastercycler epgradient S (Eppendorf, Hamburg, Germany). Each reaction contained 0.8 U Taq DNA polymerase (Thermo Fisher Scientific), 2 ng/mL *Citrus* DNA, 0.2 mm well RED (Sigma‐Aldrich, Gillingham, UK) dye‐labelled forward primer, 0.2 mm non‐dye‐labelled reverse primer, 0.2 mm each dNTP, 10 ×  PCR buffer and 1.5 mm MgCl_2_ in a final volume of 10 mL. The PCR protocol was as follows: an initial denaturation step at 94 °C for 5 min followed by 40 cycles of 30 s at 94 °C, 1 minute at 50 °C or 55 °C, 45 s at 72 °C and a final elongation step of 4 min at 72 °C. Capillary electrophoresis was performed using a CEQ^™^ 8000 Genetic Analysis System (Beckman Coulter Inc., Fullerton, CA). Data were collected and analysed using GenomeLab^®^ GeXP (Beckman Coulter Inc.) version 10.0 software.

### Genome DNA analysis

To assess whether *clbvINpr‐AtFT* integrates within the plant genome, genomic DNA extracts from bark and leaves of *clbvINpr‐AtFT*‐infected *C. excelsa* plants were analysed by PCR using primers designed on different parts of the CLBV genome and on the *CiFT* gene (Table 1). The DNA synthesized was analysed by 2% agarose gel electrophoresis and GelRed staining (Biotium Inc., Hayward, CA). The DNA fragments obtained were inserted into the pGEM‐T plasmid vector (Promega Corp.) and cloned in *Escherichia coli* DH5a cells. Positive clones were sequenced.
